# Supplementary material for: Bicyclic azetidines target acute and chronic stages of Toxoplasma gondii by inhibiting parasite phenylalanyl t-RNA synthetase
Source: Nat Commun. 2022 Jan 24;13:459. doi: 10.1038/s41467-022-28108-y (PMC8786932; doi:10.1038/s41467-022-28108-y)
Supplement: Supplementary file 1 — Supplementary Information [file 41467_2022_28108_MOESM1_ESM.pdf]

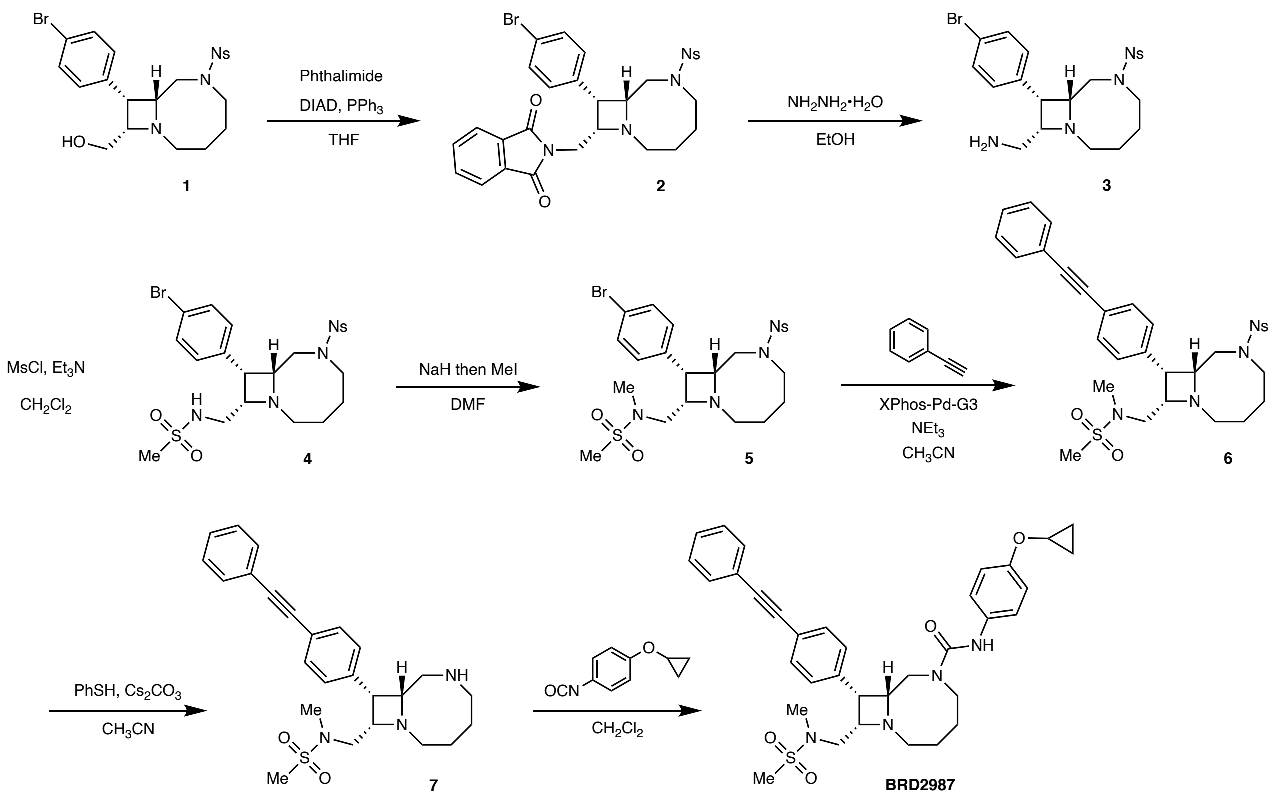

Fig. S1 Chemical synthesis of BRD2987.

Oxygen and/or moisture sensitive reactions were carried out in oven or flame-dried glassware under nitrogen atmosphere. All reagents and solvents were purchased and used as received from commercial vendors or synthesized according to cited procedures. Yields refer to chromatographically and spectroscopically pure compounds, unless otherwise stated. Flash chromatography was performed using 20-40 µm silica gel (60-Å mesh) on a Teledyne Isco Combiflash Rf. Analytical thin layer chromatography (TLC) was performed on 0.2 mm or 0.25 mm silica gel 60-F plates and visualized by UV light (254 nm). ^{1}H NMR spectra were recorded at 300 K on a Bruker 400 spectrometer (400 MHz). ^{13}C NMR spectra were recorded at 298 K on a Bruker AV-III HD spectrometer (101 MHz) equipped with a 5-mm BBO-F cryoprobe. Chemical shifts are reported in parts per million (ppm) relative to the appropriate solvent. Data for ^{1}H NMR are reported as follows: chemical shift, multiplicity (br = broad, s = singlet, bs = broad singlet, d = doublet, t = triplet, m = multiplet), coupling constants, and integration. Tandem liquid chromatography/mass spectrometry (LCMS) was performed on a Waters 2795 separations module equipped with a 3100 mass detector, alternatively a Shimadzu LC-20AD separations module or Agilent 1200 series, with data acquired either directly on reaction mixtures or on purified samples. High-resolution mass spectra (HRMS) were recorded using a Waters Acquity system equipped with a Waters Xevo G2-XS QToF mass detector.

2-(((8R,9S,10S)-9-(4-bromophenyl)-6-((4-nitrophenyl)sulfonyl)-1,6-diazabicyclo[6.2.0]decan-10-yl)methyl)isoindoline-1,3-dione (2)

To a solution of alcohol **1** (prepared as described previously[1], 2.90 g, 5.68 mmol, 1.00 equiv), phthalamide (1.00 g, 6.82 mmol, 1.20 equiv) and PPh_3 (2.23 g, 8.52 mmol, 1.50 equiv) in THF, was added dropwise DIAD (1.72 g, 8.52 mmol, 1.50 equiv) at 0°C. The mixture was stirred at 50°C for 16 hours. Next, the resulting mixture was concentrated *in vacuo* and partially purified by column chromatography (SiO_2, ethyl acetate/ petroleum ether =1:20 to 1:1) to afford a crude yellow solid (3.60 g). This material was engaged in the next step without further purification.

LC-MS m/z calculated for $C_{29}H_{28}BrN_4O_6S$ $[M+H]^+$: 641.09; observed: 641.00.

((8R,9S,10S)-9-(4-bromophenyl)-6-((4-nitrophenyl)sulfonyl)-1,6-diazabicyclo[6.2.0]decan-10-yl)methanamine (3)

Protected phthalimide **2** (3.60 g, 5.63 mmol, 1.00 equiv) was dissolved in EtOH (50 mL). $NH_2NH_2{\bullet}H_2O$ (564 mg, 11.3 mmol, 2.00 equiv) was added and the mixture was stirred at 70°C for 1 hour. After completion, the mixture was poured into water (50 mL), and then extracted with CH_2Cl_2 (3 x 50 mL). The combined organic layers were dried over Na_2SO_4, filtered and concentrated *in vacuo*. The resulting mixture was partially purified by column chromatography (SiO_2, ethyl acetate/ petroleum ether =1:20 to 1:1) to afford a brown oil (3.00 g, crude). This material was engaged in the next step without further purification.

LC-MS m/z calculated for $C_{21}H_{26}BrN_4O_4S$ $[M+H]^+$: 511.08; observed: 511.00.

N-(((8R,9S,10S)-9-(4-bromophenyl)-6-((4-nitrophenyl)sulfonyl)-1,6-diazabicyclo[6.2.0]decan-10-yl)methyl)methanesulfonamide (4)

Amine **3** (2.90 g, 5.69 mmol, 1.00 equiv) was dissolved in CH_2Cl_2 (30 mL). Et_3N (1.15 g, 11.4 mmol, 2.0 equiv) and MsCl (782 mg, 6.83 mmol, 1.20 equiv) were added at 0 °C under N_2. The mixture was stirred at 20°C for 30 minutes. After completion, the reaction was quenched by addition of ice-water (20 mL), and then extracted with CH_2Cl_2 (3 x 50mL). The combined organic layers were dried over Na_2SO_4, filtered and concentrated *in vacuo* to afford a crude yellow solid (3.76 g). This material was engaged in the next step without further purification.

LC-MS m/z calculated for $C_{22}H_{28}BrN_4O_6S_2$ $[M+H]^+$: 589.06; observed: 589.00.

N-(((8R,9S,10S)-9-(4-bromophenyl)-6-((4-nitrophenyl)sulfonyl)-1,6-diazabicyclo[6.2.0]decan-10-yl)methyl)-N-methylmethanesulfonamide (5)

Sulfonamide **4** (3.50 g, 5.96 mmol, 1.00 equiv) was dissolved in DMF (20 mL) and NaH (357 mg, 8.94 mmol, 60% w/w in mineral oil, 1.5 equiv) was added at –20 °C. The mixture was allowed to warm at 10 °C and stirred for 30 minutes. The solution was cooled down to –20 °C and MeI (1.69 g, 11.9 mmol, 2.00 equiv) was added. Then, the reaction mixture was allowed to warm at 10 °C and stirred for 16 hours. Next, the mixture was poured into cold sat. aq. NH_4Cl (20 mL) and extracted with EtOAc (3 × 30 mL). The combined organic layers were washed with brine (20 mL), dried over $MgSO_4$ and concentrated *in vacuo*. The resulting mixture was partially purified by column chromatography (SiO_2, EtOAc/petroleum ether = 1:20 to 2:1) to afford a crude yellow oil (3.00 g). A portion of this material was engaged in the next reaction without further purification.

LC-MS m/z calculated for $C_{23}H_{30}BrN_4O_6S_2$ $[M+H]^+$: 603.08; observed: 602.90.

N-methyl-N-(((8R,9S,10S)-6-((4-nitrophenyl)sulfonyl)-9-(4-(phenylethynyl)phenyl)-1,6-diazabicyclo[6.2.0]decan-10-yl)methyl)methanesulfonamide (6)

A sealed vial containing aryl bromide **5** (40.0 mg, 66.4 μmol, 1.00 equiv) was evacuated and backfilled with N_2 (×3) then were added CH_3CN (0.670 mL, previously sparged with argon for 40 min), NEt_3 (37.1 μL, 0.266 mmol, 4.00 equiv) and phenylacetylene (36.5 μL, 0.332 mmol, 5.00 equiv), followed by XPhos-Pd-G3 (5.62 mg, 6.64 μmol, 0.100 equiv). The vial was sealed and heated to 70 ºC. After 90 min, the reaction was allowed to cool at room temperature, sat. aq. $NaHCO_3$ was added, and the

mixture was extracted with CH_2Cl_2 (3 × 0.4 mL). The combined organic layers were dried over Na_2SO_4, filtered and concentrated *in vacuo*. The resulting mixture was purified by column chromatography (SiO_2, EtOAc/hexane = 0:1 to 1:1) to afford Compound **8** (16.0 mg, yield: 38%).
LC-MS m/z calculated for $C_{31}H_{35}N_4O_6S_2$ $[M+H]^+$: 623.20; observed: 623.70. **^{1}H NMR** (400 MHz, Chloroform-*d*) δ 7.82 – 7.74 (m, 1H), 7.66 – 7.41 (m, 8H), 7.35 (t, *J* = 3.3 Hz, 4H), 3.92–3.79 (m, 1H), 3.78 – 3.53 (m, 3H), 3.42 – 3.21 (m, 2H), 3.19 – 2.86 (m, 4H), 2.66 (s, 3H), 2.62 – 2.42 (m, 4H), 2.00 – 1.81 (m, 3H), 1.81 – 1.59 (m, 1H).

N-methyl-N-(((8R,9R,10S)-9-(4-(phenylethynyl)phenyl)-1,6-diazabicyclo[6.2.0]decan-10-yl)methyl)methanesulfonamide (7)
Sulfonamide **6** (30.8 mg, 49.4 µmol, 1.00 equiv) was dissolved in CH_3CN (617 µL). Cs_2CO_3 (261 mg, 80.3 µmol, 1.62 equiv) and benzenethiol (2.45 mg, 22.2 µmol, 2.27 µL, 0.450 equiv) were then added in one portion and the mixture was heated to 40 °C. After 2 h, the reaction was quenched by addition of H_2O (1 mL) and then extracted with CH_2Cl_2 (3 × 1 mL). The combined organic layers were dried over $MgSO_4$, filtered and concentrated *in vacuo*. The resulting mixture was purified by column chromatography (SiO_2, EtOAc/petroleum ether = 1:0 then CH_2Cl_2/methanol = 8:2) to afford compound **9** (10.9 mg, yield: 50%).
LC-MS m/z calculated for $C_{25}H_{32}N_3O_2S$ $[M+H]^+$: 438.22; observed: 438.29. **^{1}H NMR** (400 MHz, Chloroform-*d*) δ 7.65 – 7.46 (m, 4H), 7.46 – 7.32 (m, 5H), 3.67 (t, *J* = 7.7 Hz, 1H), 3.56 (q, *J* = 6.9 Hz, 1H), 3.44 (q, *J* = 7.2 Hz, 1H), 3.29 (dd, *J* = 14.5, 4.6 Hz, 1H), 3.16 (d, *J* = 12.0 Hz, 1H), 3.03 – 2.83 (m, 2H), 2.83 – 2.70 (m, 1H), 2.66 (s, 3H), 2.50 (s, 3H), 2.37 (dd, *J* = 14.5, 5.2 Hz, 1H), 2.24 (t, *J* = 11.5 Hz, 1H), 2.14 – 1.96 (m, 1H), 1.94 – 1.77 (m, 2H), 1.58 – 1.32 (m, 2H), 1.28 (s, 1H).

(8R,9R,10S)-N-(4-cyclopropoxyphenyl)-10-((N-methylmethylsulfonamido)methyl)-9-(4-(phenylethynyl)phenyl)-1,6-diazabicyclo[6.2.0]decane-6-carboxamide (8, BRD2987)
Amine **7** (19.8 mg, 45.2 µmol, 1.00 equiv) was dissolved in CH_2Cl_2 (2.65 mL). Et_3N (12.6 µL, 90.4 µmol, 2.00 equiv) and 4-cyclopropoxyphenyl isocyanate (prepared as described previously[2], 15.8 mg, 90.4 µmol, 2.00 equiv), were added at 0 °C under N_2. The mixture was stirred at 15 °C for 30 min and concentrated *in vacuo*. The residue was purified by flash column chromatography reverse phase (CH_3CN/water + 0.1% TFA = 0:1 to 1:1) to afford BRD2987 (23.8 mg, yield: 86%).
HRMS m/z calculated for $C_{35}H_{41}N_4O_4S$ $[M+H]^+$: 613.2843; observed: 613.2872. **^{1}H NMR** (400 MHz, Chloroform-*d*) δ 7.61 – 7.44 (m, 6H), 7.41 – 7.32 (m, 3H), 7.24 (s, 1H), 6.96 (d, *J* = 8.9 Hz, 2H), 6.07 (s, 1H), 3.85 (d, *J* = 15.6 Hz, 1H), 3.75 – 3.63 (m, 3H), 3.63 – 3.51 (m, 2H), 3.37 (dd, *J* = 14.5, 4.8 Hz, 1H), 3.27 (t, *J* = 12.8 Hz, 1H), 3.11 (t, *J* = 10 Hz, 1H), 2.98 – 2.82 (m, 2H), 2.65 (s, 3H), 2.53 (s, 3H), 2.46 – 2.35 (m, 1H), 1.93 – 1.74 (m, 3H), 1.73 – 1.59 (m, 1H), 1.26 (s, 1H), 0.74 (d, *J* = 4.4 Hz, 4H). **^{13}C NMR** (101 MHz, Chloroform-*d*) δ 131.62, 131.41, 130.92, 128.40, 122.11, 122.05, 115.17, 89.92, 88.98, 66.96, 66.57, 58.20, 50.94, 50.52, 49.03, 48.87, 43.64, 35.85, 35.13, 28.05, 27.53, 6.21.

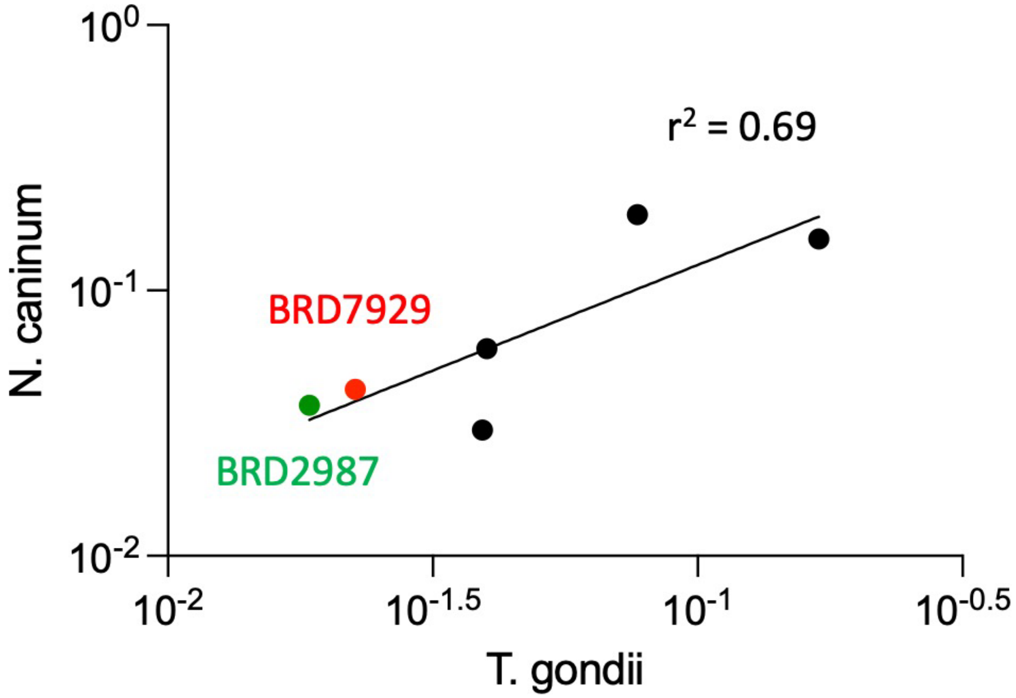

Fig. S2: Correlation of EC_{50} values for *T. gondii* and *N. caninum.*

Linear regression analysis identified a significant correlation in the growth inhibitory activities (EC_{50} values) against *T. gondii* and *N. caninum* for a subset of 6 compounds ($r^2 = 0.69$, $P = < 0.05$). Lead compounds identified against *T. gondii* highlighted: BRD2987 (green) and BR7929 (red). See Table S3 for EC_{50} values for all bicyclic azetidines tested for both parasites. Source data are provided as a Source Data file.

```
                                          Pf M316I
Pf      KNEEYKKYDIKKYNFFSSGKKINKGNIHLLTKQMRTFKEIFFSLGFEEMETHNYVESSFW 342
Pv      KNEEYKKYQVKKYNFFSSGKKMNKGNIHLLIRQMRTFKDVFVSLGFEEMNTHNYVESSFW 336
Tg      IGDAWEKSNFKEYNFFAAGKRIRRGAVHPLMQVMKQFKQILYCMGFEEMPTNQYVESSFW 289
Cp      MNSSWENNEFKPYNFNAKGKRLPRGNIHPLTRTSRKFKRILSQMGFEEMPTNRWVESSFW 267
Nc      IGDAWEKSNFKEYNFFAAGKKIRRGTVHPLMQVMKQFKQILHCMGFEEMPTNQYVESSFW 278
Hs      SSGSWRDRPFKPYNFLAHGVLPDSGHLHPLLKVRSQFRQIFLEMGFTEMPTDNFIESSFW 257
                                        Tg V262A

Pf      CFDALYIPQQHPSRDLQDTFFIKEPETCIDKF-VDTEYIDNIKRVHTHGDYGSFGWNYKW 401
Pv      CFDALYIPQQHPSRDLQDTFFIKVPEMCQEEF-TDQSYIENVKRVHSVGDYGSFGWNYQW 395
Tg      CFDSLFMPQQHPARDVQDTFFLQAPKSSDAT-KIPQAYFNSVKNIHERGGHGSIGWQYEW 348
Cp      NFDALFQPQKHPARDSHDTFFLETPSTFRNEMELSNDHIDKVKKVHEVGGYGSIGLSYNW 327
Nc      CFDALFMPQQHPARDVQDTFFLQAPKSSDAA-KIPQAYFNSVKDIHEHGGHGSIGWQYEW 337
Hs      NFDALFQPQQHPARDQHDTFFLRDPAEA---LQLPMDYVQRVKRTHSQGGYGSQGYKYNW 314

Pf      KLEESKKNVLRTHTTANSCRALFKLAKEYKEA------GCIKPKKYFSIDRVFRNENLDS 455
Pv      ELKSTKKNVLRTHTTANSCRALFQLAKEYQKT------GSIIPKKFFSIDRVFRNENLDS 449
Tg      SEEESMGNILRTHTTASSARMLYGLAQEYKKT------GVFRPRKFFSIDRVFRNETLDA 402
Cp      SIEEASKNLLRTHTTAVSVRMLYKLAQMYLYNENGLSFDNFQRKAYFSIDRVFRNESIDA 387
Nc      SQDESLGNILRTHTTASSARMLYELAQEYK------------------------------ 367
Hs      KLDEARKNLLRTHTTSASARALYRLAQKK----------PFTPVKYFSIDRVFRNETLDA 364
                                                            Pf G512E
Pf      THLAEFHQVEGLIIDKNIGLSHLIGTLAAFYKHIGIHKLKFKPTFNPYTEPSMEIYGYHE 515
Pv      THLAEFHQVEGLIIDRNLGLSHLIGTLSAFYKYIGISKLRFKPTFNPYTEPSMEVYGYHE 509
Tg      THLAEFHQVEGLVADRGLTIGHLMGVMETFYKQIGIEQLKFKPAYNPYTEPSMEIFGYHA 462
Cp      THLAEFHQVEGLIVDKNLTLADLIGTLKTFYEKIGISDLKFKPAFNPYTEPSMEIFGYHT 447
Nc      -------KVEGLVADRGLTIGHLMGVMETFYKQIGIEQLKFKPAYNPYTEPSMEIFGYHA 420
Hs      THLAEFHQIEGVVADHGLTLGHLMGVLREFFTKLGITQLRFKPAYNPYTEPSMEVFSYHQ 424

                      Tg M484I  Pf V545I  Pf L550V
Pf      QSKKWLEVGNSGIFRPEMLRSMGFSEEVSVIAWGLSLERPTMIKYNIKNIRDLFGYKSVV 575
Pv      ENKKWLEVGNSGIFRPEMLRAMGFPPEVSVIAWGLSLERPTMIKYSIRNIRDLFGYRSVL 569
Tg      GLKRWIEVGNSGIFRPEMLLPMGLPEDVTVIAWGLSLERPTMIRYGISNIRQLFGHRALL 522
Cp      TLKKWIEVGNSGLFRPEMLRPLGFPSDIVVIAWGLSLERPTMISYNIPNIRDLFSFKAKI 507
Nc      GLKRWIEVGNSGIFRPEMLLPMGLPEDVTVIAWGLSLERPTMIRYGISNIRQLFGHRALL 480
Hs      GLKKWVEVGNSGVFRPEMLLPMGLPENVSVIAWGLSLERPTMIKYGINNIRELVGHKVNL 484
                                          Tg L497I
```

Fig. S3 Alignment of PheRS sequences from human and apicomplexan parasites.

Multiple protein sequence alignment was done using Clustal Omega [3] with default settings.

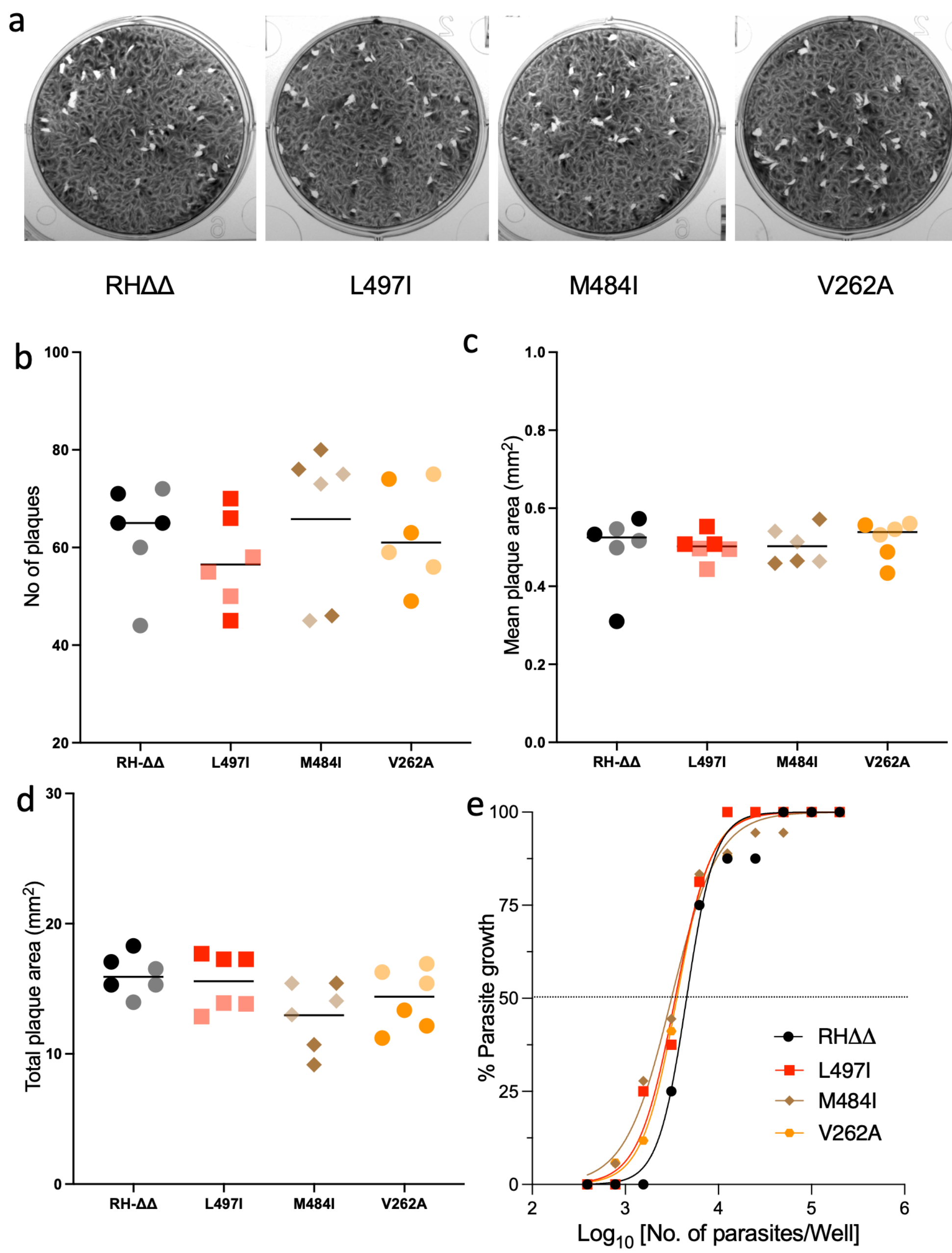

Fig. S4 Growth kinetics of BRD7929 resistant mutants and the wild type parental strain.

a) Plaque assays for TgPheRS$^{[L497I]}$, TgPheRS$^{[M484I]}$, TgPheRS[$^{V262A]}$ (denoted by the residue numbers) and parental RHΔΔ parasites grown for 7 days in HFF monolayers. Monolayers were stained with crystal violet. b-d). Quantification of total number of plaques (b), mean plaque area (c), and total plaque area (d) showed no significant differences between the four parasites. Two biological replicates are shown, each with three technical replicates (dark and shaded symbols denote separate experiments) with the median represented by the black line. Two-way ANOVA with Sidak's correction for multiple comparisons. e) Lytic assay for growth of parasites on HFF monolayers showed similar growth curve for the resistant mutants and parental strain. Growth curves are based on two biological replicates each with three technical replicates. Source data are provided as a Source Data file.

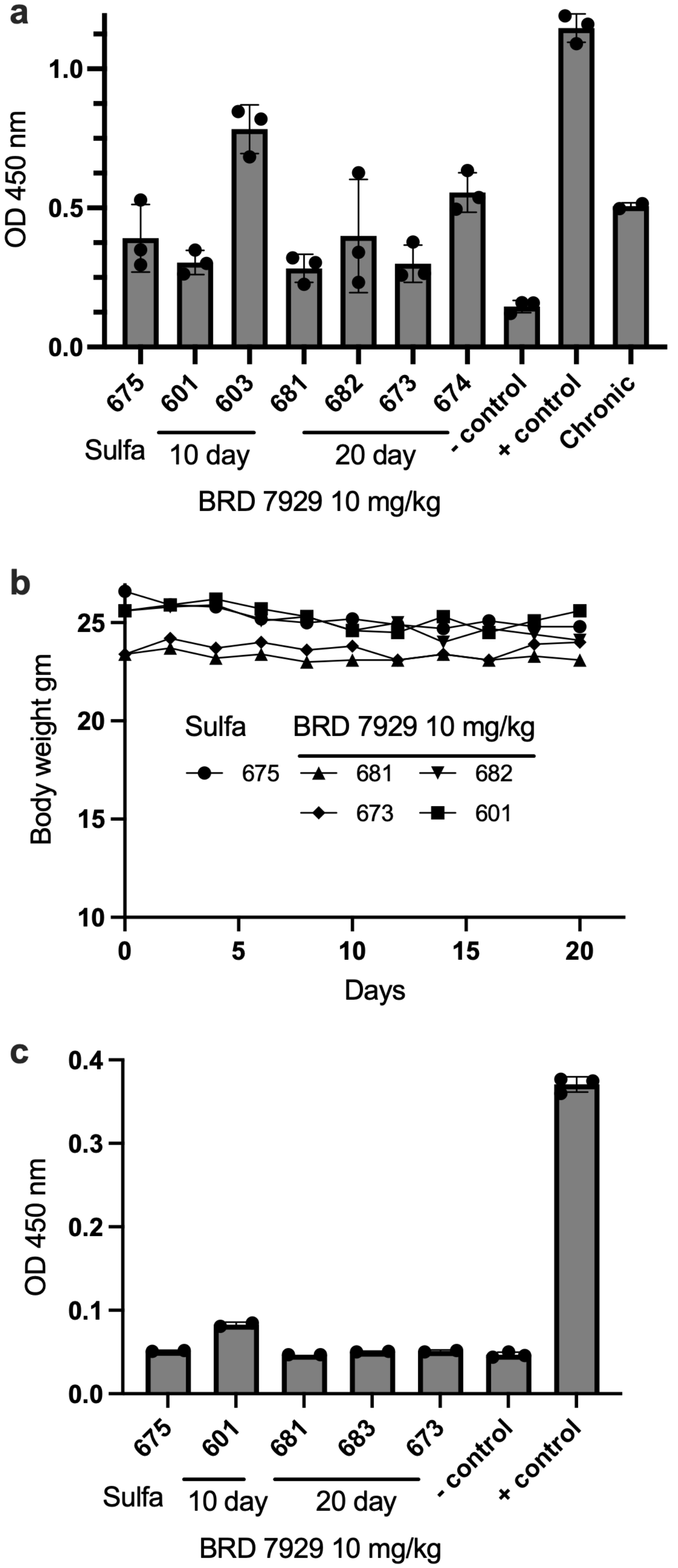

Fig. S5 ELISA and survival data for in vivo efficacy trials. a) ELISA data for animals in the efficacy trial shown in Fig. 6c. Surviving animals at 30 days post infection were tested by ELISA to confirm infection. – control represents pooled sera from naïve animals. + control represents pooled sera from chronically infected animals that were not part of this trial. Prior to bioassay, two animals succumbed to infection (# 603 and 674). Animal numbers listed on the X ordinate. Values are means ± S.D. for three technical replicates. The animal listed as "chronic" represents the sole surviving animal from the trial in Fig. 6e. b) Surviving animals from A were sacrificed, the brains removed and homogenized, and 20% of the volume was orally gavaged into one recipient Ifngr1$^{-/-}$ mouse for each donor. The body weight of the recipient animals was followed for 20 day in the absence of treatment and none of them became sick. c) ELISA values for the recipient animals in the bioassay shown in b. Recipient animals are denoted by the animal number of the donor animal. – control represents pooled sera from naïve animals. + control represents pooled sera from chronically infected animals that were not part of this trial. Values are means ± variance for two technical replicates. Source data are provided as a Source Data file.

Table S1: EC_{50} values for growth inhibition of *T. gondii* tachyzoites (ME49-FLuc) by bicyclic azetidines.

Broad ID	short ID	rank order	*T. gondii* EC_{50} (μM)
BRD-K78162987-001-02-3	BRD2987	1	0.018
BRD-K78727929-001-03-2	BRD7929	2	0.022
BRD-K83953444-001-02-5	BRD3444	3	0.028
BRD-K21118494-001-01-1	BRD8494	4	0.034
BRD-K63655018-001-03-4	BRD5018	5	0.039
BRD-K38642419-001-01-3	BRD2419	6	0.039
BRD-K70383882-001-01-1	BRD3882	7	0.050
BRD-K10803316-001-01-7	BRD3316	8	0.064
BRD-K80013914-001-01-4	BRD3914	9	0.076
BRD-K35212132-003-01-0	BRD2132	10	0.089
BRD-K37749995-001-02-7	BRD9995	11	0.118
BRD-K91490185-001-01-3	BRD0185	12	0.169
BRD-K74624873-001-01-5	BRD4873	13	0.213
Pyrimethamine	pyr	14	0.247
BRD-K04066479-001-01-2	BRD6479	15	0.424
BRD-K82568493-001-01-4	BRD8493	16	0.441
BRD-K73045999-001-01-3	BRD5999	17	0.492
BRD-K77399257-001-01-7	BRD9257	18	0.646
BRD-K73572574-001-01-9	BRD2574	19	1.487
BRD-K84092430-001-01-9	BRD2430	20	1.548
BRD-K55645774-001-01-3	BRD5774	21	1.609
BRD-K73462936-001-01-6	BRD2936	22	1.687
BRD-K48893182-001-01-5	BRD3182	23	1.697
BRD-K99759599-001-01-3	BRD9599	24	1.748
BRD-K11928260-001-01-7	BRD8260	25	2.575
BRD-K15888104-001-01-9	BRD8194	26	2.955
BRD-K06904569-001-01-3	BRD4569	27	3.376
BRD-K89925349-001-01-5	BRD5349	28	3.944
BRD-K10113801-001-01-3	BRD3801	29	4.096

Table S2: Parasite strains and genotypes of *T. gondii* generated for this study.

**Genotype**	**genetic lineage**	**clone ID Reference**
GT1*; uprt::TUB:FLUC,* ^{fl}*DHFR*^{fl}	I	A
RH; *uprt::TUB:FLUC,* ^{fl}*DHFR*^{fl}	I	4
ME49-FLuc	II	Tobin and Knoll 2012[4]
CTG; *uprt::TUB:FLUC,* ^{fl}*DHFR*^{fl}	III	B
MAS; *uprt::TUB:FLUC,* ^{fl}*DHFR*^{fl}	IV	B2
RUB; *uprt::TUB:FLUC,* ^{fl}*DHFR*^{fl}	V	H1
FOU; *uprt::TUB:FLUC,* ^{fl}*DHFR*^{fl}	VI	G2
VAND; *uprt::TUB:FLUC,* ^{fl}*DHFR*^{fl}	X	F6
RH-B2	I	Rosenberg et al 2019[5]
RH-B2; *uprt::TUB:FLUC,* ^{fl}*DHFR*^{fl}	I	F1
RH*Δhgxprt**Δku80*	I	Huynh and Carruthers 2009[6]
RH*Δhgxprt**Δku80;* phers$^{[V262A]}$	I	1
RH*Δhgxprt**Δku80;* phers$^{[M484I]}$	I	3
RH*Δhgxprt**Δku80;* phers$^{[L497I]}$	I	20
RH*Δhgxprt**Δku80;* phers$^{[L497VI]}$	I	47
RH*Δhgxprt**Δku80;* phers$^{[V2626A +M484I]}$	I	E4

Table S3: EC_{50} values of bicyclic azetidines against *C. parvum* and the veterinary pathogen *N. caninum.*

Broad ID	short ID	*C. parvum* EC_{50} (μM)	*N. caninum* EC_{50} (μM)
BRD-K10803316-001-01-7	BRD3316	0.016	
BRD-K99759599-001-01-3	BRD9599	0.25	
BRD-K73462936-001-01-6	BRD2936	1.08	
BRD-K91490185-001-01-3	BRD0185	0.033	
BRD-K80013914-001-01-4	BRD3914	0.062	
BRD-K83953444-001-09-0	BRD3444	0.015	
BRD-K63655018-001-03-4	BRD5018		0.029
BRD-K78162987-001-02-3	BRD2987		0.037
BRD-K80013914-001-01-4	BRD3914		0.193
BRD-K91490185-001-01-3	BRD0185		0.156
BRD-K78727929-001-03-2	BRD7929		0.042
BRD-K38642419-001-01-3	BRD2419		0.060

Table S4: Comparison of IC_{50} for TgPheRS enzymes *vs.* EC_{50} for inhibition of *T. gondii* growth

TgPheRS	EC_{50}[a]	IC_{50}[b]
Wild type	0.036	0.031
L497V	1.423	2.108
M484I	0.065	0.135
L497I	2.312	1.315
V262A	0.159	0.207
V262A-M484I	0.234	0.197

[a] Monitored by growth restriction, Average value from n = 3 biological replicates
[b] Monitored by enzyme inhibition, Average value from n = 3 biological replicates
Linear regression analysis of IC_{50} vs. EC_{50} $r^2 = 0.92$

References

1. Lowe, J.T. et al. Synthesis and profiling of a diverse collection of azetidine-based scaffolds for the development of CNS-focused lead-like libraries. *J Org Chem* **77**, 7187-7211 (2012).
2. Sharma, M. et al. Structural basis of malaria parasite phenylalanine tRNA-synthetase inhibition by bicyclic azetidines. *Nat Commun* **12**, 343 (2021).
3. Sievers, F. et al. Fast, scalable generation of high-quality protein multiple sequence alignments using Clustal Omega. *Mol Syst Biol* **7**, 539 (2011).
4. Tobin, C.M. & Knoll, L.J. A patatin-like protein protects *Toxoplasma gondii* from degradation in a nitric oxide-dependent manner. *Infect Immun* **80**, 55-61 (2012).
5. Rosenberg, A., Luth, M.R., Winzeler, E.A., Behnke, M. & Sibley, L.D. Evolution of resistance in vitro reveals mechanisms of artemisinin activity in *Toxoplasma gondii*. *Proc Natl Acad Sci U S A* (2019).
6. Huynh, M.H. & Carruthers, V.B. Tagging of endogenous genes in a *Toxoplasma gondii* strain lacking Ku80. *Eukaryot Cell* **8**, 530-539 (2009).
