# Supplementary material for: Bicyclic azetidines target acute and chronic stages of Toxoplasma gondii by inhibiting parasite phenylalanyl t-RNA synthetase
Source: Nat Commun. 2022 Jan 24;13:459. doi: 10.1038/s41467-022-28108-y (PMC8786932; doi:10.1038/s41467-022-28108-y)
Supplement: Supplementary file 5 — Reporting Summary [file 41467_2022_28108_MOESM5_ESM.pdf]

nature portfolio

Corresponding author(s): L. D. Sibley

Last updated by author(s): Jan 7, 2022

Reporting Summary

Nature Portfolio wishes to improve the reproducibility of the work that we publish. This form provides structure for consistency and transparency in reporting. For further information on Nature Portfolio policies, see our Editorial Policies and the Editorial Policy Checklist.

Statistics

For all statistical analyses, confirm that the following items are present in the figure legend, table legend, main text, or Methods section.

n/a	Confirmed	
☐	☒	The exact sample size (n) for each experimental group/condition, given as a discrete number and unit of measurement
☐	☒	A statement on whether measurements were taken from distinct samples or whether the same sample was measured repeatedly
☐	☒	The statistical test(s) used AND whether they are one- or two-sided<br>*Only common tests should be described solely by name; describe more complex techniques in the Methods section.*
☒	☐	A description of all covariates tested
☐	☒	A description of any assumptions or corrections, such as tests of normality and adjustment for multiple comparisons
☐	☒	A full description of the statistical parameters including central tendency (e.g. means) or other basic estimates (e.g. regression coefficient) AND variation (e.g. standard deviation) or associated estimates of uncertainty (e.g. confidence intervals)
☐	☒	For null hypothesis testing, the test statistic (e.g. F, t, r) with confidence intervals, effect sizes, degrees of freedom and P value noted<br>*Give P values as exact values whenever suitable.*
☒	☐	For Bayesian analysis, information on the choice of priors and Markov chain Monte Carlo settings
☒	☐	For hierarchical and complex designs, identification of the appropriate level for tests and full reporting of outcomes
☒	☐	Estimates of effect sizes (e.g. Cohen's d, Pearson's r), indicating how they were calculated

Our web collection on statistics for biologists contains articles on many of the points above.

Software and code

Policy information about availability of computer code

Data collection	Cytation 3 multi-mode imager (BioTek); NovaSeq6000 (Illumina) ; InCell Analyzer 2000 (GE Healthcare); AxioObserver (Zeiss); Dual Pod FX with SAMI Workstation EX v4.1 (Biomek); SpectraMax M2 (Molecular Devices); Combiflash Rf (Teledyne ISCO); Waters 2795 separations module (Waters); Waters 3100 mass detector (Waters); 1200 series HPLC (Agilent); LC-20AD separations module (Shimadzu); Bruker 400 NMR (Bruker)
Data analysis	Prism version 9 (Graphpad), Excel 365 v16, 2016(Microsoft), CLC Genomics Workbench v20 (Qiagen), InCell Developer Software package vl.9 (GE Healthcare); Gen 5 v3.08 (BioTek); Prime v4.0 (Schrodinger); Phoenix WinNonlin v6.3 (Certara); MestReNova vl2.0.4-22023 (Mestrelab Research), Clustal Omega v1.2, Chimera vl.13.1, ImageJ v1.53a.

For manuscripts utilizing custom algorithms or software that are central to the research but not yet described in published literature, software must be made available to editors and reviewers. We strongly encourage code deposition in a community repository (e.g. GitHub). See the Nature Portfolio guidelines for submitting code & software for further information.

Data

Policy information about availability of data

All manuscripts must include a data availability statement. This statement should provide the following information, where applicable:

- Accession codes, unique identifiers, or web links for publicly available datasets
- A description of any restrictions on data availability
- For clinical datasets or third party data, please ensure that the statement adheres to our policy

The datasets for whole genome sequencing generated during the current study are available in the short read archive (SRA) of NCBI under the accession number PRJNA731915 (https://www.ncbi.nlm.nih.gov/sra/?term=PRJNA731915). PDB files generated in previous studies and used here include PDB 3L4G (https://

www.rcsb.org/structure/3l4g) and PDB 2IY5 (https://www.rcsb.org/structure/2IY5). All other data are found in the paper, in the supplementary information files, or source data that are provided with this paper. Unique materials described in this report are available under standard Material Transfer Agreements that can be arranged by contacting the corresponding author.

Field-specific reporting

Please select the one below that is the best fit for your research. If you are not sure, read the appropriate sections before making your selection.

- [x] Life sciences
- [ ] Behavioural & social sciences
- [ ] Ecological, evolutionary & environmental sciences

For a reference copy of the document with all sections, see nature.com/documents/nr-reporting-summary-flat.pdf

Life sciences study design

All studies must disclose on these points even when the disclosure is negative.

Sample size	Samples sizes were chosen to provide an estimate of the sample variance (to determine if parametric vs non-parametric statistical tests were appropriate) and to support appropriate statistical testing for difference in population means or distributions.
Data exclusions	none
Replication	All experiments were completed two or more times to ensure similar results between replicates and specific replicate details for biological and technical replicates described within each associated figure legend
Randomization	Samples used for control or treatment groups were chosen at random. For animal experiments, groups were chosen to distirubte different sexes across control and treated groups.
Blinding	Samples were not blinded as the outcomes of all assays were quantitative rather than subjective.

Reporting for specific materials, systems and methods

We require information from authors about some types of materials, experimental systems and methods used in many studies. Here, indicate whether each material, system or method listed is relevant to your study. If you are not sure if a list item applies to your research, read the appropriate section before selecting a response.

Materials & experimental systems

n/a	Involved in the study	
[ ]	[x]	Antibodies
[ ]	[x]	Eukaryotic cell lines
[x]	[ ]	Palaeontology and archaeology
[ ]	[x]	Animals and other organisms
[x]	[ ]	Human research participants
[x]	[ ]	Clinical data
[x]	[ ]	Dual use research of concern

Methods

n/a	Involved in the study	
[x]	[ ]	ChIP-seq
[x]	[ ]	Flow cytometry
[x]	[ ]	MRI-based neuroimaging

Antibodies

Antibodies used	mouse mAb 8.25.8 anti-BAG1
Validation	Produced by the laboratory of Louis Weiss. Validated by immunofluorescence staining of in vitro produced bradyzoites.

Eukaryotic cell lines

Policy information about cell lines

Cell line source(s)	HepG2 (human hepatocellular carcinoma (ATCC-HB-8065)), THP-1 (human monocytic tumor line), SH-SY5Y (human neuroblastoma (ATCC CRL-2266)), A549 (human lung carcinoma (ATCC CRM-CCL-185)) and Caco-2 (human intestinal adenocarcinoma (ATCC HTB-37) were obtained from ATCC and HFF (primary human foreskin fibroblast) obtained from the laboratory of John Boothroyd at Stanford University.
Authentication	Cells obtained from ATCC were used within 5 passages of receipt and were not further authenticated. HFF cells retain their fibroblast morphology and express markers consistent with their lineage over 20+ passages.
Mycoplasma contamination	Cell culture samples were tested and confirmed negative for mycoplasma.

Commonly misidentified lines (See ICLAC register)	*Name any commonly misidentified cell lines used in the study and provide a rationale for their use.*

Animals and other organisms

Policy information about studies involving animals; ARRIVE guidelines recommended for reporting animal research

Laboratory animals	CD-1 female mice aged 8-12 weeks (Charles River Lab); male and female Ifngr1-/- (B6.129S7-Ifngr1 tm1Agt /J) and CBA/J mice aged 8-12 weeks (Jackson Laboratory); C57B/6 female mice aged 8-12 weeks (Jackson Laboratory). Animals were maintained on a 12:12 light cycle, room temperature maintained at 70F +/- 2F, and room humidity maintained at 50% +/- 20%.
Wild animals	none
Field-collected samples	none
Ethics oversight	Animal studies were conducted according to the U.S. Public Health Service policy on human care and use of laboratory animals. Animals were maintained in facilities approved by the Association for Assessment and Accreditation of Laboratory Animal Care. Studies were approved by Division of Comparative Medicine, Washington University.

Note that full information on the approval of the study protocol must also be provided in the manuscript.
